# Extracellular Vesicles and Purinergic Signaling in Alzheimer’s Disease—Joining Forces for Novel Therapeutic Approach

**DOI:** 10.3390/brainsci15060570

**Published:** 2025-05-26

**Authors:** Julita Lewandowska, Jakub Majewski, Katarzyna Roszek

**Affiliations:** Department of Biochemistry, Faculty of Biological and Veterinary Sciences, Nicolaus Copernicus University in Torun, Lwowska 1, 87-100 Torun, Poland

**Keywords:** neurodegenerative diseases, extracellular vesicles, purinergic signaling, P2X receptors, neuroinflammation

## Abstract

Neurodegenerative diseases, including Alzheimer’s disease (AD), are a global problem affecting millions of people. Thanks to years of research and huge efforts, it has been possible to discover the pathophysiological changes accompanying Alzheimer’s disease at the cellular level. It turns out that the formation of amyloid-beta plaques and hyperphosphorylation of tau protein in the brain play a key role in disease development. Purinergic signaling (PS) is implicated in the pathophysiology of several disorders in the central nervous system, and recent findings link some disturbances in PS with Alzheimer’s disease. The primary objective of our review is to comprehensively explore and identify key purinergic signaling targets that hold therapeutic potential in the treatment of patients suffering from the disease. In particular, we focus on the dual role of purinergic compounds and extracellular vesicles (EVs), which have emerged as critical components in cellular communication and disease modulation. The extracellular vesicles that are naturally released by various cells fulfill the role of communication tools, also by harnessing the purinergic compounds. In this context, our review presents a thorough and integrative analysis of how extracellular vesicles can influence purinergic signaling and how this interaction might be leveraged to develop novel, targeted treatment strategies. Ultimately, this line of research may lead to innovative therapeutic approaches that are not only effective in slowing or halting disease progression but also demonstrate a high degree of biocompatibility and safety for the human organism.

## 1. Introduction

### 1.1. Alzheimer’s Disease

Alzheimer’s disease (AD) is one of the most prevalent neurodegenerative diseases, a slowly progressive central nervous system (CNS) disorder responsible for most dementia cases. People affected by this disease suffer a gradual loss of memory, difficulties in language and reasoning, behavioral cognitive impairment, and eventually basic bodily malfunctions. As a result, they suffer the inability to function independently, a gradual loss of self-care ability, and major mental disability, ultimately resulting in a serious decrease in AD patients’ life quality. The disorder has become a major public health problem, bringing a heavy burden to individuals, society, and families, considering the fact that AD patients require constant care from caregivers or family [1,2,3].

The disease was described as “presenile dementia” first in 1906 by German psychiatrist and neuropathologist Alois Alzheimer. One year later, the famous Alzheimer’s paper was published, which for the first time in history described the case of a patient affected by the disease bearing the name of the scientist to this day [4,5]. According to the epidemiology update of Alzheimer’s Disease International from 2023, the number of people suffering from the disorder was estimated to be around 55 million worldwide in 2019, and this number was predicted to triple by 2050, giving a total of approximately 139 million patients [6]. The World Health Organization’s (WHO) data from 2017 indicate that the risk of AD development increases with age [7]. The newest data indicate that 11% of people over the age of 65 are affected by the disease, while among patients over the age of 85, it rises to a striking percentage of 42% [8]. Moreover, the incidence of the disease is estimated to be higher for women than for men [7].

Despite many studies, the causes of AD are not fully recognized but are likely to include a combination of genetic, age, environmental, and lifestyle factors. Scientists report that during AD progression, the disorder is divided into the early stages and the long pre-symptomatic phase, which can last up to 20 years, further complicating early diagnosis. To this day, there is no effective cure available to stop the development of AD, although there are special lifestyle recommendations that are said to slightly lower the risk of the disorder’s occurrence. The goal of a majority of scientists is to identify and provide the therapy that can delay the disease progression [7,8].

### 1.2. Mechanism of the Disorder Development

Alzheimer’s disease, as a neurodegenerative disorder, causes progressive loss of brain neurons, associated with dysfunction of synapses and neuronal networks. Neuronal loss occurs gradually and is related to the degree of gray matter loss, correlating with the stage of AD advancement [9,10,11,12,13,14,15]. There are several different hypotheses of the genesis of Alzheimer’s disease. The first, so-called cholinergic hypothesis, appearing in the literature for the longest time, suggests that AD is caused by an imbalance of the neurotransmitter acetylcholine as a result of reduced biosynthesis. The second theory, one of the most frequently cited and best scientifically proven, is the amyloid hypothesis, which assumes that the formation of amyloid-beta (Aβ) plaques is mainly responsible for the impairment of neurons in AD. Some of the existing theories describe an additional role of tau protein, which may also be crucial in the development of the disorder [16,17].

Amyloid-β monomer is a small peptide (up to 43 amino acids long) naturally secreted as a part of proper synaptic activity. The monomer Aβ1–40 is a product of the enzymatic cleavage of transmembrane amyloid precursor protein (APP) involving specific secretases (α, β, and γ) [17,18]. Even though the function of both APP protein and Aβ peptide in nervous cells is not particularly known, it is confirmed that mutations in the APP coding gene may affect the structure of the protein, possibly leading to cleavage pattern changes. Following further, those changes may lead to the synthesis of extracellular Aβ isoform, longer by two amino acids (isoleucine and alanine), resulting in a molecule of 42 amino acids in total length. Aβ1–42 is highly associated with AD development; more Aβ42 molecules change the Aβ42/Aβ40 ratio, contributing to the formation of deposits of Aβ [10,19,20]. Aβ42 isoform is more likely to aggregate into the form of variably structured soluble neurotoxic oligomers [21], then insoluble fibrils, and finally amyloid plaques. Amyloid-β peptide plaques deposit in parts of the brain and are recognized as foreign material, causing an inflammatory and immune response [9,16,17,18,19,20,22]. Additionally, the impaired processes of Aβ42 elimination contribute to the increased production of reactive oxygen species (ROS), pro-inflammatory cytokines, tumor necrosis factor-alpha (TNF-α), chemokines, and increase the release of nucleotides, eventually contributing to cell death [23].

**Figure 1:**
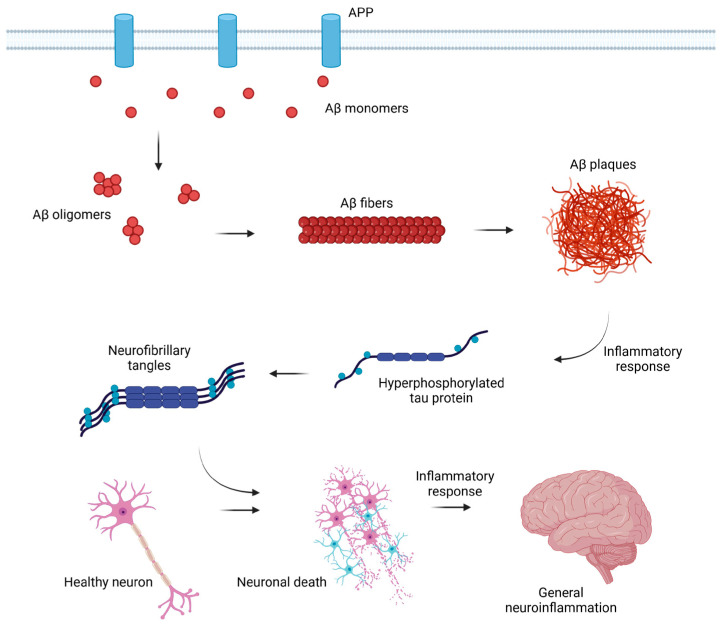
Development of neuroinflammation in AD. Amyloid-beta monomers are formed as a result of the transmembrane APP cleavage by secretases. Monomers group into more complex structures, which are oligomers, fibrils, and plaques. Formation of plaques results in the initiation of an inflammatory response, which initiates hyperphosphorylation of tau protein. Tau protein is detached from microtubules, and neurofibrillary tangles are formed, which have a toxic effect on the nervous cells. Healthy neurons become diseased and finally undergo degradation, which is another important factor initiating inflammation in the central nervous system. Created in BioRender: Lewandowska, J. (2025), https://BioRender.com/edmwusi, based on [9,28].

Aβ isoforms activate the cascade of reactions, eventually leading to hyperphosphorylation of another important factor in AD etiology, tau protein. Tau is a neuronal microtubule-associated protein that ensures microtubule (MT) polymerization, thus maintaining the integrity and correct structure of the cytoskeleton of brain neurons, enabling proper axonal transport [24,25]. Under physiological conditions, tau protein binds to MTs through phosphorylation of serine/threonine carried out by a variety of kinases: A-kinase, C-kinase, cyclin-dependent kinase-5 (CDK-5), CaM kinase II, Fyn kinase, glycogen synthase kinase-3β (GSK-3β), and MAPKs, whereas the binding itself can occur at binding sites located at the N-terminal region, repeat region, and C-terminal region. Under pathological conditions, on the other hand, tau protein’s binding sites are being hyperphosphorylated. The process of hyperphosphorylation is caused by the overactivity of serine/threonine kinases, such as CDK-5 and GSK-3β, through deregulation of the kinase stimuli (such as p53) by Aβ aggregates [17,26,27]. The hyperphosphorylation eventually leads to the detachment of tau protein from MTs, causing not only disturbances in the structure and functioning of brain neurons but also increasing the risk of the formation of neurotoxic tau oligomers and intracellular neurofibrillary tangles (NFTs) [17,26]. The immune response to this situation can result in the release of inflammatory mediators by microglia and astrocytes and finally exacerbate the chronic neuroinflammation of AD patients [8]; see Figure 1.

Many in vitro and in vivo studies have shown that the formation of Aβ plaques and NFTs is crucial in the process of AD development, which ultimately leads to the destruction of brain neuron structures, resulting in cognitive impairment and memory loss deeply associated with Alzheimer’s disease [29]. In addition, Aβ affects cerebral vasoconstriction, which is most likely caused by the formation of free radicals. Disturbances can have fatal consequences, elevating blood pressure and even leading to cerebral hypoxia [30].

Due to the importance of these two molecules, they are commonly called AD markers. The majority of the histopathological markers of the AD brain can be detected postmortem. The tau pathology spreads throughout the brain in a predictable, characteristic pattern. On the other hand, amyloid plaques display a less predictable spreading pattern, whereby their presence does not correlate well with cognitive decline either. Although the exact spreading mechanism of Aβ oligomers is not completely understood, extracellular vesicles (EVs) have been proposed as one of the contributors [10]. Their role will be discussed in further chapters.

### 1.3. Basics of Neuroinflammation in AD

The inflammatory theory of AD pathogenesis assumes the contribution of activated microglia and reactive astrocytes. Interestingly, inflammation occurs before protein markers of the disease (misfolded proteins) appear. Acute inflammation in the nervous system is a key process that helps protect the brain from pathogens [25,26,27,28,29]. It is associated with the release of pro-inflammatory cytokines, chemokines, and also growth factors participating in adenosine-mediated neurorepair, which stimulates cell proliferation and angiogenesis [30,31,32,33,34,35,36]. When the process becomes chronic, inflammation-based astrocyte or microglial dysfunction may lead to the formation of plaques and NFTs [37,38,39,40,41]. Additionally, Aβ aggregates enhance the prolonged chronic activation of microglia, a process characterized by the further release of pro-inflammatory cytokines, neurotoxins, and free radicals, which cause degeneration of neurons [42,43]. On the other hand, in the early stages of AD, it has been shown that inflammation may be neuroprotective and activation of microglia by pro-inflammatory cytokines may increase the clearance of neurotoxic Aβ and dead neurons, resulting in enhanced brain homeostasis and synapse stability [44,45].

The sustained neuroinflammation can be detrimental, perpetuating a cycle of neuronal damage and degeneration. Both activated microglia and astrocytes mutually enhance further inflammation by impairing their ability to promote neuronal survival, growth, synaptogenesis, and phagocytosis [46,47,48,49,50,51,52,53,54,55]. Cells participating in the CNS immune response can detect Aβ through toll-like receptors, leading to the increased production of ROS and pro-inflammatory cytokines (e.g., interferon-gamma (IFN-γ), interleukins: IL-1β, IL-6, and TNF-α), which further contribute to neuronal death and axonal damage [56,57,58]. The damaged neurons release inter alia significant amounts of nucleotides, which participate in the progression of the disease and, on the other hand, in inducing neuroprotective mechanisms. β-amyloid itself is also a strong factor that causes the release of adenosine triphosphate (ATP) by astrocytes and microglia. Extracellular nucleotides, with ATP as a key inflammatory mediator, participate in the development of inflammation, among others, by inducing microglial chemotaxis [59], the release of cytokines (IL-1β, IL-6, TNF-α) [58,60], and astrogliosis, which is a well-described feature of AD [61]. The presence of extracellular nucleotides under physiological conditions is precisely controlled by the activity of ecto-enzymes, and any disturbances in this dynamic balance can contribute to a variety of impairments. That also implies a complex role of purinergic signals in Alzheimer’s disease-affected neurons [44,62,63,64,65].

## 2. Purinergic Signaling in CNS and Its Role in Alzheimer’s Disease Development

The purinergic signaling concept goes back to the year of 1972 when neurobiologist Geoffrey Burnstock indicated that the molecule of ATP not only plays a role as a cellular energy carrier but also is an important factor in extracellular signalization. This statement not only changed the way the ATP molecule itself was viewed but also launched a completely new direction of research [63,66]. After many years, it is known that not only adenosine triphosphate (ATP) but also adenosine diphosphate (ADP), adenosine monophosphate (AMP), and adenosine (Ado) play important roles in the purinergic signaling pathway [64]. Enzymes called ectonucleotidases are responsible for maintaining purine and pyrimidine homeostasis and creating their appropriate concentrations in the extracellular environment. Extracellular ATP and ADP can be hydrolyzed to AMP by extracellular nucleotidase CD39 (ecto-nucleoside triphosphate diphosphohydrolase 1—NTPDase 1) and other NTPDases. In turn, AMP can be hydrolyzed to adenosine by another extracellular nucleotidase—CD73 (ecto-5′-nucleotidase-5′-NT). Adenosine can be metabolized to inosine by adenosine deaminase or rephosphorylated to AMP by adenosine kinase. Adenylate kinase, alkaline phosphatases, and ectonucleoside pyrophosphatases/phosphodiesterases also play a role in these fine-tuned metabolic processes [64,65,67,68].

Nucleotides and nucleosides are recognized by specific receptors commonly distributed in mammalian cells. Purinergic receptors were isolated, cloned, and characterized into two main classes, depending on their agonist selectivity: nucleoside P1R (with adenosine as a natural ligand) and nucleotide P2R (with ATP and/or ADP as natural ligands). Adenosine P1 receptors were divided into four subtypes: A1, A2A, A2B and A3, mainly depending on their structure and interactions with different members of G protein family (stimulating or inhibiting) [69], while P2 receptors were divided into two subtypes: P2X and P2Y, with, respectively, seven (P2X1-7) and eight (P2Y1, 2, 4, 6, 11–14) members of these subgroups [65,69,70].

It is commonly known that extracellular nucleosides and nucleotides play the role of intercellular messengers. In the central nervous system, their role is extended to participation in neurotransmission, modulation of sensory stimuli, and induction of an immune response in the event of pathological conditions. Considering the role of purinergic signaling in the CNS, it is not surprising that it has been shown to be involved in the processes of initiation and progression of Alzheimer’s disease [30,71]. Each of the molecules involved in PS has its own function, but the most important in maintaining the physiological state and developing the pathological state are considered to be adenosine and ATP [30,72].

### 2.1. Adenosine and Its Receptors

Numerous studies show that adenosine is continuously formed under physiological conditions both intracellularly and in an extracellular environment [68]. Normally present in the CNS extracellular fluid at nanomolar concentrations, ranging from 30 to 300 nM, Ado exerts neuroprotective effects. Moreover, it is known that adenosine in an appropriate concentration acts as a pro-proliferation signal, indicating a physiological environment. Bearing that in mind, numerous compounds in CNS cells increase the potential of endogenous adenosine and enable its maintenance at an appropriate concentration. These include inhibitors of enzymes involved in the process of adenosine degradation or transformation, e.g., ADA (adenosine deaminase) inhibitors and AKA (adenosine kinase) inhibitors, as well as those regulating Ado transport to the extracellular environment [30,72]. Of the four subtypes of adenosine receptors, A1 and A2A are most highly expressed in the central nervous system cells, whereas A2B and A3 receptors are characterized by much lower expression within this area [30,72,73]. Signal transduction via all adenosine receptors occurs directly by coupling with heteromeric G protein complexes (composed of a Gα subunit and a heterodimeric Gβγ subunit). Since adenosine is not stored in the synaptic vesicles and is released to the cytoplasm through nucleoside transporters, it cannot be treated as a classical neurotransmitter. Nevertheless, its influence on neurotransmission modulation as an extracellular signal molecule is undeniable and crucial for keeping the homeostasis and proper function of the nervous system [74]. Signaling cascades involving post-activation reactions of adenosine receptors have different downstream effects depending on the cells that are being stimulated (Table 1). In the case of neuronal cells, this action will be based on stimulation or inhibition of neurotransmitter secretion, as well as control of neuronal excitability. It is worth mentioning that P1 receptors play a key role not only in neuronal activity but also in coordinating the function of cells as astrocytes and glial cells, which is crucial in the precise control of CNS homeostasis and maintaining inflammatory balance [75].

**Figure 2:**
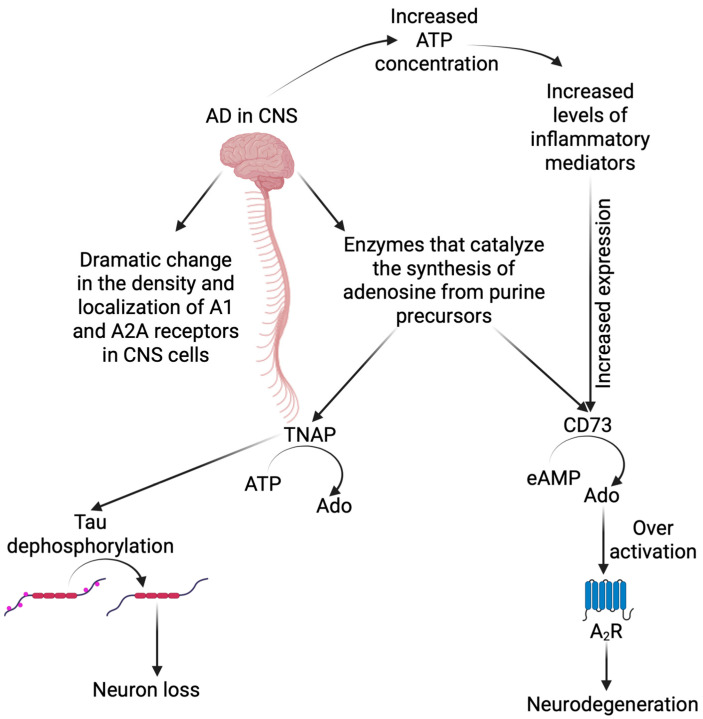
Changes in the purinergic system in Alzheimer’s disease. During the course of Alzheimer’s disease, the release of ATP outside the cells via lytic and nonlytic mechanisms occurs in the central nervous system. Activation of P2X7 receptor enhances inflammatory processes. The enzyme TNAP (tissue nonspecific alkaline phosphatase), involved in the degradation of ATP to adenosine, may also contribute to neurotoxicity by dephosphorylating tau protein. In turn, ecto-5′-nucleotidase (CD73) is responsible for the conversion of AMP to adenosine. Ado production leads to activation of A_2_A receptors, the excessive stimulation of which intensifies neurodegenerative processes. Created in BioRender. Lewandowska, J. (2025), https://biorender.com/0ci7425.

In terms of Alzheimer’s disease, the important feature of adenosine A1 and A2A receptors is the fact that their density and localization in the CNS cells, especially in the frontal cortex, undergo drastic changes demonstrated both in vivo studies using a mouse AD model, and in postmortem AD patients’ samples; see Figure 2. Moreover, adenosine homeostasis is also disturbed [75,81]. Postmortem analyses of the brains of Alzheimer’s patients and PET (positron emission tomography) studies have shown a significant decrease in the expression level of A1 receptors in the dentate gyrus and hippocampus, as well as in the temporal cortex, i.e., areas of NFT spread and, consequently, neuronal degeneration [75,82,83]. Studies conducted on mouse models of AD have shown that in animals with mild tau pathology, it was possible to reverse it with a simultaneous decline in cognitive functions by intravenous administration of a selective A1 receptor antagonist (rolofylline). However, in animals with advanced tau pathology, the process could not be reversed. Thus, the inhibition of receptor activity was pivotal to stopping the progression of disease changes. The authors explain the results by the stimulation of adenylyl cyclase through the lack of A1 receptor activity, and therefore by an increase in the level of cyclic AMP, which in turn activates kinase A, stimulating the system supporting the removal of unfolded proteins, which includes tau [84]. Moreover, the researchers also found a relationship between the level of A2A receptor expression in the brains and peripheral blood platelets of Alzheimer’s patients. A significant increase in the level of receptors in the CNS cells was associated with an increase in A2AR expression on platelets in patients, compared to the control group [85]. Studies have also shown an inverse relationship between frequent and regular caffeine consumption and the risk of dementia, including AD. Caffeine, as a non-selective A1 and A2A receptor antagonist, may be involved in reducing the toxicity of Aβ plaques in cultured rat and mouse neurons, as well as preventing the formation of NFTs [86,87]. It is also known that adenosine receptors indirectly affect synaptic plasticity (e.g., by causing prolonged excitation or inhibition), which is a positive effect in the search for potential anti-AD therapies [30].

Tissue nonspecific alkaline phosphatase (TNAP), a CNS enzyme that participates in the degradation of ATP to adenosine, has been found to be associated with neuronal toxicity through mechanisms involving tau dephosphorylation and may cause neuronal loss in AD [88,89]. The association between another protein belonging to the ecto-enzymes group, namely ecto-5′-nucleotidase, which converts AMP to adenosine, and the A2A receptor has solid molecular support [90,91]. It has been shown to be functionally important for controlling synaptic plasticity under physiological conditions, as well as in morpho-functional changes in animal models of neurodegenerative diseases such as Alzheimer’s disease [92]. The CD73-mediated dephosphorylation of extracellular AMP (eAMP) to Ado acts as the main control point for extracellular Ado levels and results in the activation of adenosine A2A receptors. Excessive activation of this receptor significantly increases the mechanisms associated with neurodegeneration, while genetic and pharmacological blockade of A2AR provides solid neuroprotection [93,94]; see Figure 2. It is worth noting that the signal for increased CD73 expression is a state of hypoxia and elevated levels of inflammatory mediators [95]. The relationships in changes in the density of P1 receptors have been proven both in animal models and in humans, and therefore, there are premises that may indicate the possibility of using changes in the expression of adenosine receptors in the diagnosis and treatment of Alzheimer’s disease [75,85]. 

### 2.2. ATP and Its Receptors

Extracellular ATP is a natural ligand for P2 group membrane receptors—ion channel P2XR (all isoforms) and G protein-coupled P2YR (mainly for P2Y1, P2Y2, and P2Y11 subtypes) [70,96,97]. The diversity of purinergic receptors, as well as the omnipresence of ATP in various concentrations within the central nervous system, means that purinergic signaling plays multiple roles in promoting the proper development and functioning of cells within the CNS, and also plays a significant role in pathological conditions [97]. Many properties of extracellular ATP enable the molecule to deliver cell-to-cell signals under pathological conditions. ATP can act as a neurotransmitter, neuromodulator, growth factor, or toxicant and is often released concurrently, e.g., with neurotransmitters, to modify physiological or pathological effects [16]. Under physiological conditions, ATP enters the extracellular space in small amounts (nanomolar range) through astrocytes and neurons, forming synaptic connections [61]. It also serves as a substrate for the enzymatic degradation to lower adenosine phosphate molecules (ADP and AMP), as well as to adenosine itself [97].

#### 2.2.1. P2X Receptors

P2X receptors, of which ATP is a key agonist, are ion channels permeable to Na^+^, K^+^, and Ca^2+^ ions. The best described mechanism of activation of these receptors by ATP causes an increase in intracellular Ca^2+^ concentration, which induces depolarization and activates multiple signaling pathways, including MAPK (mitogen-activated protein kinase), protein kinase C (PKC), calcineurin, or NFi-κβ (nuclear factor κβ) [61,73,98]. The most abundant P2X receptors in CNS are listed in Table 2.

Although the expression of all P2X receptor subunits has been demonstrated in the CNS, the best described and most promising in the fight against Alzheimer’s disease seems to be the P2X7 receptor [61]. This isoform is highly expressed in the immune effector cells, such as monocytes, macrophages, and T-cells, as well as in astrocytes, oligodendrocytes, and neurons [61,99]. P2X7R differs significantly from the others in terms of ATP sensitivity. The level of the receptor-activating molecule must be as high as 100–1000 μM, considered a concentration that occurs only under pathological conditions, while in the case of other P2X receptors, a nanomolar to low-micromolar concentration is sufficient. Prolonged activation of the P2X7 receptor leads to transient pore formation in cell membranes. Under pathological conditions, ATP activates microglia via P2X7 receptors, causing increased release of interleukins (IL-1β, IL-6, IL-10), TNF-α, transforming growth factor-β (TGF-β), and cytotoxic levels of glutamate [100,101].

**Table 2:** Selected examples of P2X receptors widely expressed in the CNS, along with the receptor activation effect.

Purinergic Receptor	Placement in the CNS	Receptor Activation Effect	Reference
P2X_1_	Postsynaptic neurons	Interneuron depolarization	[102]
P2X_2_	Presynaptic neurons	Increase in glutamate release	[102]
P2X_3_	Presynaptic neurons	Interneuron depolarization	[103]
P2X_4_	Microglia	Increase in migration and secretion	[104]
P2X_7_	Microglia	Activation of inflammasome	[105,106]
	Leukocytes	Necrotic cytolysis Apoptotic death	[99]
	Oligodendrocytes	Promotion of demyelination	[107]

Numerous studies conducted on both animals and humans clearly indicate the involvement of the P2X7 receptor in Alzheimer’s disease. The relationship between tau protein and the level of P2X7R expression was assessed. In mouse models, inhibition of receptor expression was shown to reduce the rate of protein phosphorylation and the formation of deposits of its incorrectly folded form in intraneuronal areas of the hippocampus [108,109]. In mouse models with increased P2X7R expression, increased tau protein pathology was observed, which confirms the hypothesis of an unequivocal effect of the receptor on pathologies characteristic of Alzheimer’s disease [108]. Studies conducted on transgenic mouse models (developing amyloid plaques) of Alzheimer’s disease have shown a significant increase in the expression of this receptor at both the mRNA and protein levels, thus indicating the influence of Aβ-plaques on the stimulating regulation of receptor expression [101]. Neurodegenerative Aβ aggregates are formed from amyloid precursor protein after cleavage by β- and γ-secretases, in contrast to the soluble APP fragment (sAPPα) generated by α-secretase, which has a neuroprotective effect [106,110]. Activated P2X7 receptor also contributes to the reduced α-secretase activity [110].

In AD, not only are Aβ aggregates accumulating in the cells problematic, but abnormalities of α-synuclein (ASN), one of the main components of protein deposits (Lewy bodies), have been abundantly observed. ASN can enter the extracellular space via exocytosis, where it changes conformation to a β-sheet structure, and then, this structure penetrates neuronal or glial cells, contributing to neurodegeneration and neuronal death [111,112]. Stimulation of the microglial P2X7 receptor with extracellular ASN increases oxidative stress, mitochondrial dysfunction, and ROS production, and may contribute to neurodegenerative mechanisms [112,113]. Therefore, it has been concluded that the use of P2 receptor antagonists may have a neuroprotective effect and effectively reduce the formation of amyloid plaques [112]. Similarly, the activation of P2Y2 receptors may have a neuroprotective effect opposite to activated P2X7R [30].

#### 2.2.2. P2Y Receptors

Metabotropic P2Y receptors are divided into P2Y1, P2Y2, P2Y4, P2Y6, P2Y11–14, each of which is expressed in CNS cells such as neurons, astrocytes, microglia, and oligodendrocytes [61]. What distinguishes the P2Y receptor group from the others is the fact that P2YRs are sensitive to both purine and pyrimidine nucleotides. Specific for adenine nucleotides are: P2Y1—ADP and ATP, P2Y11—ATP, P2Y12—ADP, P2Y13—ADP and ATP, while P2Y4, P2Y6, and P2Y14 are receptors for uracil nucleotides (UTP or UDP) [30,61,114]. Similarly to adenosine P1 receptors, this type of receptor also couples with G proteins [115]. Each group binding to a different G protein has a different second messenger system. P2Y1, P2Y2, P2Y4, and P2Y6 receptors, which are coupled with Gq protein, increase the intracellular Ca^2+^ concentration and activate the protein kinase C when ligand bound, whereas P2Y12–14 receptors couple with Gi/o protein to decrease the cyclic AMP (cAMP) production by inhibiting adenylate cyclase when activated. P2Y11 receptor can couple with both Gq and Gs proteins, and its activation leads to the release of Ca^2+^ but also an increase in adenylyl cyclase activity, which stimulates the production of cAMP [115,116]. In the central nervous system, these receptors play a role in neuroinflammation, neurotransmission, and neurogenesis as well as in neuroprotective processes, including contributing to the degradation of neurotoxic Aβ [117].

In both rat cerebral cortex cells and microglia, Aβ1–42 causes the release of ATP outside the cells via one of two mechanisms: lytic or nonlytic [29,55]. P2Y2 activation on migrating microglial cells increases the uptake of fibrillar Aβ1–42 and oligomeric Aβ1–42. Unfortunately, the neuroprotective role of P2Y2 occurs only in the initial phase of Alzheimer’s disease, i.e., up to 25 weeks in the mouse model of AD. It is related to the fact that during the progression of Alzheimer’s disease, the expression of this receptor decreases, which intensifies the progression of the disease [30,62,117]. It is worth noting that P2Y receptors are responsible for controlling both the production and removal of Aβ [62].

Purinergic signaling can also effectively reduce the intensity of the immune response by regulating the P2Y1R and P2Y2R [118]. Inflammation is crucial for increased expression and activation of the P2Y2 receptor, which can exert neuroprotective effects only after activation [119]. During the progression of Alzheimer’s disease, the expression of the P2Y2 receptor is reduced, meanwhile, the expression of the P2Y1 receptor, sensitive to ADP, is increased. Interestingly, P2Y1 receptor expression was found to be highest in astrocytes around amyloid plaques [120]. Moreover, in the brains of Alzheimer’s patients, a correlation was demonstrated between the localization of P2Y1 receptors and neurofibrillary tangles, as well as Aβ plaques [62]. P2Y1 receptor blockade has been found to normalize astroglial network dysfunction, and modulation of this receptor may also help alleviate inflammation and cognitive decline [120]. The potential importance of P2Y1 receptors in Alzheimer’s disease is supported by studies conducted in vitro on mouse neural stem cell (NSC) cultures, which showed that activation of these receptors by ADP stimulates the development of NSCs. Since neuronal degradation is one of the hallmarks of neurodegenerative diseases, the results of these studies indicate the therapeutic potential of P2Y receptors in the fight against Alzheimer’s disease [62].

### 2.3. Main Players in Purinergic Signaling in AD

#### 2.3.1. Astrocytes

Astrocytes, belonging to the CNS cells, play an extremely important role in protecting the brain through regulated communication with other cellular elements of the nervous tissue, including neurons and microglia. During Alzheimer’s disease, astrocytes, as a result of interactions with Aβ plaques, undergo transformations and become “reactive”, eventually losing their function. Astrocytes also demonstrate the ability to internalize modified forms of tau protein, contributing to the propagation of this protein [121,122]. During AD, astrogliosis also occurs, a process aimed at minimizing brain damage. It can therefore be considered that both of these processes may constitute a nonspecific marker of Alzheimer’s disease, as they occur not only in the course of Alzheimer’s disease but also in acute and chronic neuronal damage [120]. Astrocytes are cells capable of secreting ATP under pathological conditions via astrocytic connexin hemichannels. Then, the released adenosine triphosphate (ATP) can be converted to adenosine by ectonucleotidases [121]. Astrocytes have surface receptors that enable them to recognize molecules, e.g., those released after cell damage, including ATP, pathological Aβ, and tau species [123,124,125,126,127]. Ligand and receptor binding of astrocytes enables a rapid response to a pathological condition, including the initiation of inflammation. Abnormal levels of ATP can enable the activation of P2X7 receptors on astrocytes, thereby contributing to impaired neurotransmission that can lead to AD [121]. Emerging studies are investigating different tactics involved in regulating this receptor, for example, in a mouse model of AD, deletion of the P2X7R receptor resulted in improved synaptic plasticity and improved learning [128].

#### 2.3.2. Microglia

Microglial cells are an extremely important element of the CNS. In an active state, they act neurotoxically or neuro-protectively, e.g., by changing their phenotype to macrophage cells capable of phagocytosis [119].

Although the M1/M2 classification of microglia is still commonly referenced, current evidence suggests that this binary paradigm oversimplifies the functional heterogeneity of microglial populations. Recent findings indicate that microglia represent a dynamic and diverse cell community comprising multiple subtypes with distinct phenotypes, gene expression profiles, and responses to various stimuli. Undoubtedly, the need for more nuanced classification systems that better reflect the complexity of microglial activation state must be highlighted [129].

Microglial cells are able to migrate in response to high concentrations of ATP and its derivatives in the extracellular space, and this process is possible due to the presence of P2Y12 and P2X4 receptors [119,130]. It has been proven that activated microglial cells, after ATP-stimulated P2Y12 receptor activation, act against Aβ plaques by endocytosing them [62,131]. This process would support the hypothesis that extracellular nucleotides serve as endogenous danger signals to activate the innate immune and defensive response [119,132,133]. On the other hand, glial cells exposed to Aβ plaques can, like astrocytes, release eATP via hemichannels that act on the P2X7 receptor, increasing inflammation [34,134]. Aβ aggregates induce microglial-mediated neuronal degeneration, and P2X7R expression on microglia has also been shown to be associated with Aβ plaques [106]. Glial cells interact with neurons and other CNS cells to maintain normal synaptic activity. Unfortunately, these interactions do not always have a positive effect on the nervous system, e.g., in the case of Alzheimer’s disease, microglia with a changed phenotype interact with neurons, contributing to the impairment of the function of neuronal circuits [119]. Physiologically normal microglia may adopt distinct disease-associated microglia (DAM) profiles in patients with Alzheimer’s disease [135,136]. This is because Aβ is recognized by receptors present on microglia, which causes their transition/activation into DAM [137]. It is worth noting that microglia are capable of phagocytosis only after transforming into the amoeboid phenotype, and only in this state do microglia release compounds such as IL-1β, TNF-α, chemokines, excitotoxic ATP, and glutamate through vesicular exocytosis, influencing the inflammatory response throughout the CNS [138,139]. Additionally, eATP, through the activation of the P2X7 receptor, suppressed the release of IL-1β by blocking tonically active potassium channel (THIK-1) [140]. THIK-1 regulates IL-1β release in response to purinergic stimulation from rodent microglia [140,141]. Inhibition of this receptor, e.g., by using antagonists, may be an effective and microglia-specific therapeutic strategy aimed at suppressing inflammation in the CNS [142].

#### 2.3.3. Extracellular Vesicles

The transmission of different signals, including purinergic ones, between cells is carried out in various ways. One of them uses heterogeneous extracellular vesicles (EVs). Initially, vesicle release was thought to be primarily a process allowing proper cells to get rid of waste materials to maintain homeostasis or for cancer cells to promote tumor progression and metastasis [143]. However, it is now known that it plays a crucial role in intracellular communication. Vesicles secreted by one cell are taken up by another [143,144]. Numerous studies indicate that EVs are secreted by all cells of the human body, as evidenced by the presence of vesicles in all tissues and body fluids under both physiological and pathological conditions [142]. Research has proven the unquestionable participation of extracellular vesicles in neural cell proliferation and differentiation, immune modulation and senescence, as well as the maintenance of homeostasis in the central nervous system [144,145]. EVs are structures composed of a lipid bilayer membrane that forms the vesicle envelope, separating its interior from the external environment. Vesicular cargo is highly dependent on the stage of biogenesis in which the cell is, the cell type, and its physiological state. In general, however, the interior of vesicles contains organic molecules such as proteins, lipids, and nucleic acids [146]. Due to their small size, EVs can cross the blood–brain barrier [147,148,149]. It is worth mentioning that all cells of the body secrete different types of EVs, which do not have a nucleus and, therefore, cannot replicate themselves [150]. Quick release of EVs enables effective and fast cell–cell communication over closer and further distances, and the process is possible thanks to the low immunogenicity of vesicles [147]. Extracellular vesicles are currently classified into two types based on size: microvesicles and exosomes [146].

**Figure 3:**
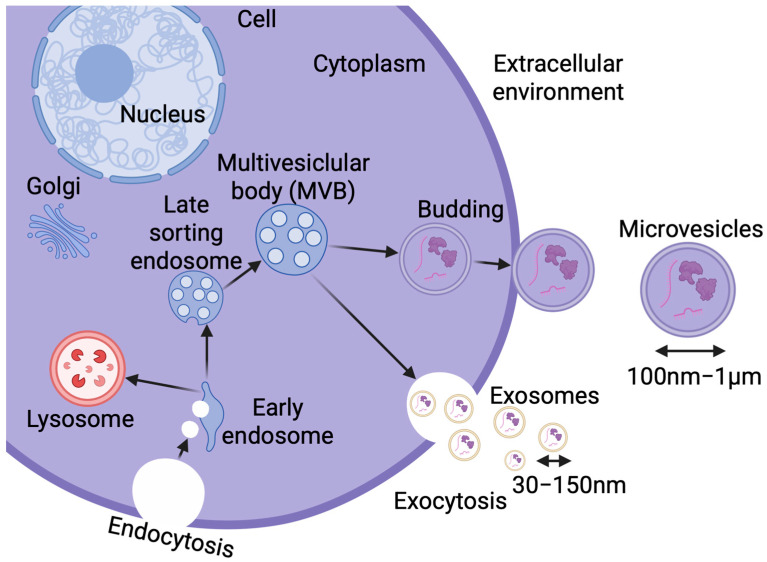
Formation and classification of extracellular vesicles (EVs). Vesicles are secreted by all cells of the human body and found in all tissues and body fluids. EVs are divided into two main types depending on their size and mechanism of formation: microvesicles and exosomes, both released into the intercellular space. Created in BioRender. Lewandowska, J. (2025), https://biorender.com/fpkc2br.

The term microvesicles is used for EVs with sizes in the range of 100 nm–1 μm, released from the cell by escape from multivesicular bodies (MVBs) and budding from the outer surface of the cell membrane [147]. The membrane of these structures is covered with biomarkers such as selectins, integrins, flotillin-2, and CD40 [151]. Exosomes, known also as tolerosomes and prostasomes, are smaller (30–150 nm) vesicles that derive from the endolysosomal pathway and the fusion of MVBs with the plasma membrane [147,152,153], and then they are released into the intercellular space [147]; see Figure 3. Exosomes may carry a variety of cargoes, including nucleic acids (microRNAs, mRNA, DNA, mitochondrial DNA, ribosomal RNA, and long non-coding RNAs), proteins (heat shock proteins, lipid-associated proteins, and cytoskeletal proteins), and lipids (lipid raft-associated lipids, ceramides, sphingolipids, phospholipids, and glycerol phospholipids) [154,155]. Both exosomes and microvesicles larger than 100nm usually contain cytoplasmic components, including mitochondrial cristae, outer and inner membranes, and also show the ability to express ATP synthase [156].

**Figure 4:**
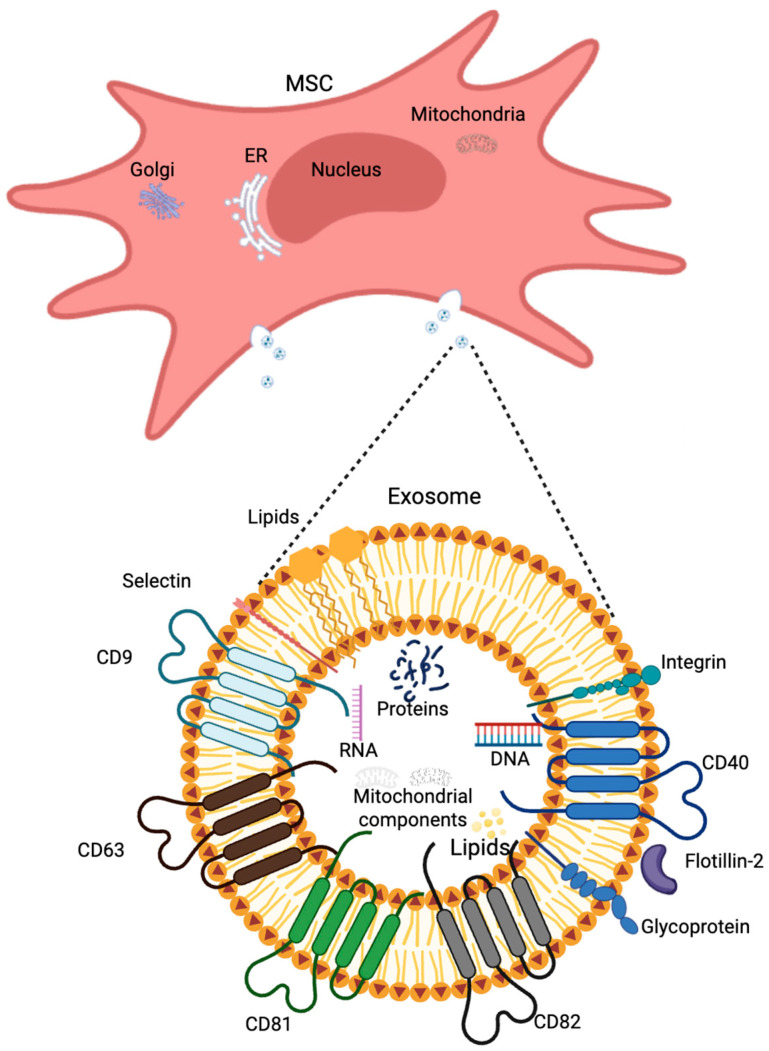
Schematic composition of extracellular vesicles (EVs) released from cells such as mesenchymal stem cells (MSCs) by exocytosis. Created in BioRender. Lewandowska, J. (2025), https://BioRender.com/kcz73yw, based on [160].

EVs play a key role in processes such as angiogenesis, inflammation [155,157], transport of morphogens, and apoptosis [158]. The molecules on their surface may differ significantly depending on the cell type, its age, and physiological state at the time of vesicle secretion, but there are individual biomarkers characteristic of most extracellular vesicles, including TSG101 (tumor susceptibility gene 101), ALIX (also known as programmed cell death 6-interacting protein), and the tetraspanins CD9, CD63, CD81 [151], CD82, adhesion proteins, integrins and glycoproteins [158]; see Figure 4. Exosomes may also carry purine nucleosides and nucleotides, as well as they may be involved in the process of adenosine synthesis from ATP due to CD39 and CD73 activity [159,160]. Treg-derived exosomes express membrane-bound and soluble CD73 enzyme isoforms, as well as AMPase. This indicates the ability to generate Ado from extracellular AMP to implement Ado activity as a regulator of the immune response [161,162].

The presence of functional mitochondrial outer and inner membranes, cristae, and mitochondrial DNA in extracellular vesicles causes cellular bioenergetics to be significantly influenced through ATP production. On the one hand, increased ATP availability can lead to the intensification of neurodegenerative processes by activating P2X7 receptors. On the other hand, the transfer of functional mitochondrial structures via EVs can limit the level of ROS and support cellular metabolism, which translates into increased survival of astrocytes, macrophages, and neurons, especially under hypoxia and glucose deprivation (OGD) conditions [156,163,164]. It is worth noting that mtDNA mutations or mitochondrial dysfunctions are often associated with neurodegenerative diseases [165,166]. The purine content of EVs is clearly related to the presence or ability to express purinergic system components, the content of which may differ between EVs depending on the cell origin. MVs derived from astrocytes show the presence of NTPDase, which is activated under conditions of oxygen and glucose deprivation [167,168].

A hot topic regarding exosomes may be the reason for their release [169,170], which includes different forms of cellular stress. Processes such as hypoxia, heat stress and chemotherapy, low or high glucose, and oxidative stress [171,172,173,174,175,176] increase exosome production. Regardless of the cause, one of the key compounds stimulating the EV release from cells is ATP [177,178]. Exosomes interact with a variety of different cell targets, and their functionally active cargos delivered to recipient cells reprogram them by surface receptor-ligand signaling or by genetically mediated transcriptional alterations after internalization [146], which can occur via phagocytosis, pinocytosis, endocytosis, or plasma membrane fusion [179]. Many studies also confirm the participation of EVs released from the tumor niche in the preparation of distant tissues of the organism for tumor metastasis [157].

## 3. Extracellular Vesicles Contribution to Alzheimer’s Disease

Extracellular vesicles are becoming the subject of many studies, where they are used as efficient vehicles for the release of signaling molecules in intercellular communication and information exchange. Scientists have reported the participation of EVs not only in physiological but also in pathological processes, an example of which is their participation in the spread of pathogenic proteins associated with neurodegenerative diseases. On the other hand, the information exchange can promote neuronal survival, synapse assembly, and plasticity [11]. It is worth noting that Aβ peptides, which are key for AD progression, accumulate inside neurons in acidic cellular compartments, including late endosomes; thus, multivesicular bodies (MVBs) are an extremely important area of the cell, binding the generation, aggregation, and accumulation of Aβ together with EVs [10]. Studies on AD also claim the possibility of transporting Aβ in its oligomeric and neurotoxic form through neurons by EVs, and thus contribute to the pathogenesis of Alzheimer’s disease [10,157]. Addition of microglia-derived microvesicles to Aβ1–42 aggregates in vitro may promote the formation of small soluble neurotoxic Aβ1–42 species, thereby increasing neurotoxicity in cultured primary hippocampal neurons [10]. Exosomes cause aggregation of Aβ protein, accumulating around neuritic plaques, causing the formation of amyloid plaques [153]. EVs are also able to transport key factors in the body, such as APP, β-site APP cleaving enzyme 1, γ-secretase and their pyrolysis products, soluble APP β, soluble APP α, CCAAT box-binding transcription factors (CTFs), and Aβ [180], while APP-related metabolites may accumulate in exosomes under AD pathogenic conditions, contributing to disease progression [181]. In addition to Aβ protein, EVs can also carry tau protein, which can also be released into the extracellular space as a “seed”. The uptake of pathological tau “seeds” leads to tau misfolding into a toxic conformation in recipient cells, causing long-distance propagation of tau pathology and neurodegeneration [153]. It was proved that tau-containing EVs have the potential to mediate the propagation of tau pathology in vitro [10,182].

On the other hand, EVs are also involved in mechanisms such as the transfer of neuroprotective substances between cells and initiate neuroprotective processes that reduce AD progression [153]. It has been shown that using EVs isolated from N2a neuroblastoma cells reduces sympathetic damage in a mouse model of AD. Such EVs can also form an EV-Aβ complex, thanks to which the incorrect protein is degraded faster by microglia in vitro. EVs isolated not only from neuroblastoma cells but also from astrocytes can affect the aggregation and reduction of the pool of incorrectly formed Aβ protein and thus reduce the progression of AD. Vesicles derived from neurons administered to the hippocampus also affect the reduction of Aβ protein levels. However, the authors of the study emphasize that the administered EVs come from healthy sources in which properly functioning microglia were present [10]. Additionally, EVs may also play a role in eliminating the inflammation of the nervous system, for example, through the transport of therapeutic agents, such as miR-124–3p. Exosomes derived from microglia transporting miR-124–3p may cause AD pathology remission, contributing to the promotion of Aβ recognition and phagocytosis by microglial cells. The mechanism of the process includes the involvement of chemokines [153].

### Therapeutic Potential of Extracellular Vesicles with Purinergic Compounds 

Limited data point out that the purinergic cargo of EVs and the activity of CD39/CD73 could be potentially beneficial for improving the nucleotide balance in the CNS disturbed under pathological conditions. However, there is an urgent need to search for such a therapeutic approach and, consecutively, for a rich source of exogenous EVs. Mesenchymal stem cells are one of the possibilities. However, there is still a lack of information on whether and how nucleotides affect the secretion of extracellular vesicles by MSCs. Studies conducted on other cell types, such as macrophages, microglia, dendritic cells, and cancer cells, indicate that ATP acting through activation of the P2X7 receptor is an efficient inducer of vesicle release [183]. The participation of EVs derived from mesenchymal stem cells (MSCs) has been recently studied as potential therapeutic agents against AD. MSCs derived from adipose tissue reduce β-amyloidosis [154] and neuronal apoptosis enhance axonal growth in a mouse model of AD [184]. EVs isolated from ADSCs were described to contain the enzyme neprilysin (NEP), which degrades Aβ, effectively reducing the level of Aβ and cell apoptosis [185,186]. One of the MSC surface antigens, 5′-nucleotidase/CD73, was also proved to be present on the EVs membrane, which confirms the capability to produce Ado and increase its concentration in the target extracellular environment [159].

**Figure 5:**
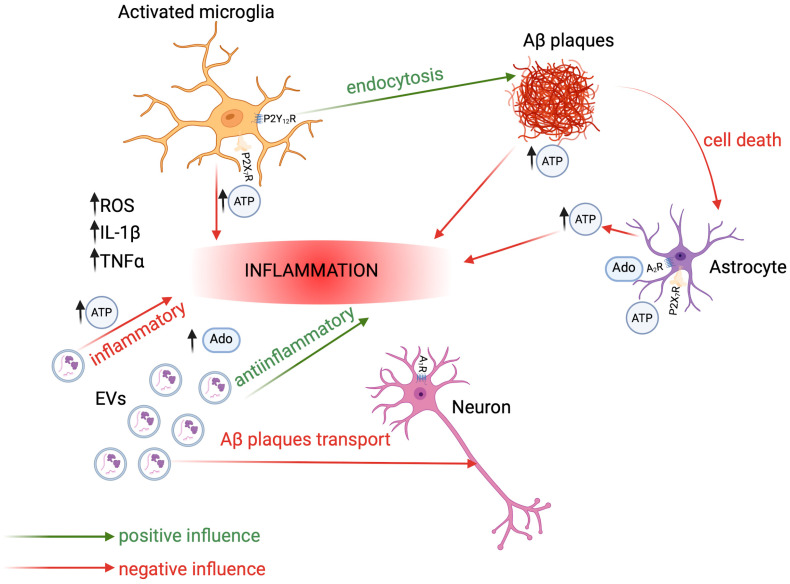
The complex picture of interactions within the diseased central nervous system. Microglia, extracellular vesicles secreted by, among others, mesenchymal stem cells, as well as cytokines and purine compounds, have the ability to modulate the functioning of CNS cells. Depending on the content of the vesicles, this action can have a neurodegenerative or neuroprotective effect on neurons. Further details are provided in the main text. Created in BioRender. Lewandowska, J. (2025), https://BioRender.com/q56o75m, based on [159,160].

In general, the EV-mediated generation of adenosine and subsequent activation of the purinergic signaling cascade is an effective mechanism to induce immunosuppression and counteract inflammatory processes [159,160]; see Figure 5. Many MSC-derived EVs were proven to transport cytokines, which allows for further inhibition of the immune system activity or induction of an anti-inflammatory response, which is another beneficial therapeutic effect [8]. MSC-derived extracellular vesicles were also reported to induce neuroprotection by modulating PI3K/Akt pathway and calcium oscillations [187]. The PI3K/Akt pathway can be aberrantly activated through various mechanisms, including purinergic receptor activation. The role of exosomes from MSCs has also been shown as a potential source of purines, primarily ATP, capable of activating microglia through P2Y receptors activation [62,132], so MSC-derived EVs can effectively reduce the pathogenesis of AD [188,189,190]. Additionally, exosomes derived from MSCs can effectively reduce the level of Aβ protein aggregates, decreasing the negative effects of this amyloid, e.g., declined cognitive functions in AD patients [153] or downregulation of synaptic plasticity-related genes [191]. The potential role of ATP-binding cassette (ABC) transporters in understanding the pathological deposition of peptides during neurodegenerative diseases has also emerged recently. Transporters have been confirmed to be involved in Aβ clearance, which constitutes a putative molecular target for the treatment of AD [192]. The final emphasis on the role of EVs during AD is the fact that, leading to the inhibition of vesicle generation, uptake, and secretion, the neurodegeneration of neuronal cells can be stopped, due to the limitation of the Aβ protein spread [10]. Thus, decreasing ecto-ATP concentration can likely be a practicable approach to limiting endogenous EV release and to counteract neuroinflammation. Although current evidence suggests that targeting purinergic cues through EVs may represent a promising therapeutic approach in AD, no registered clinical trials are investigating the role of EVs in AD.

## 4. Concluding Remarks and Future Perspectives

The great advantage of EVs as therapeutics and carriers of active substances to CNS is their ability to cross the blood–brain barrier (BBB) in a bidirectional manner, as well as their low immunogenicity [11]. EVs exhibit the homing ability and can target pathological regions in AD models, thus representing promising drug delivery platforms for AD treatment [8]. There are many ways to isolate EVs, but none of them is ideal, and it is still unknown which source of vesicles will be the best for their effective isolation [11]. Finding an effective method for isolating vesicles on a large scale is still challenging, and EVs themselves are also difficult to characterize and distinguish from each other based on size and charge, thus, isolated fractions are usually not 100% homogeneous [152]. Another problem is that the most effective route of administering therapeutic EVs has not yet been characterized [150].

Therapeutic agents directed against AD are most often based on one of three possible mechanisms of action: clearing abnormal protein accumulation, achieving neuroprotection, and immunomodulatory effects. Possibly, all these modes can be ascribed to EV’s cargo, specifically to adenosine, in terms of neuroprotection and immunomodulation. Vesicles can also be considered carriers for genetic therapies, because they can be engineered to carry and deliver active molecules, in the form of proteins, RNA, or genetic material [193]. Moreover, EVs are not capable of self-replication, which reduces the potential risk when used as a carrier/therapeutic agent. When mentioning existing therapeutic approaches, aducanumab (Aduhelm) should be mentioned, which has been approved in the United States as a drug directed against amyloid-β (Aβ) aggregates. However, this therapeutic agent, as well as other anti-AD agents, are used only to improve the quality of a patient’s life or prolong it. None of these drugs will reverse neurodegeneration, therefore, new strategies for the treatment of AD are in great demand [8].

Purinergic signaling is an extremely complex network of receptors, nucleotides, and ectonucleotidases responsible for their degradation. New therapeutic strategies may not only base their action on changing the activity of receptors, including agonists/antagonists, but also on changing their expression and manipulating the release and decay of extracellular nucleotides [61]. The latter approach seems to be very promising, specifically in regards to functional contribution of eATP to neuropathology (neuroinflammation, hyperexcitability, and neurodegeneration) and the contribution of eAdo to neuroprotective and immunomodulatory processes. Maintaining the balanced concentrations of purine nucleotides and nucleosides will allow the control of the proper cytophysiology of CNS cells.

## Figures and Tables

**Table 1:** Selected examples of adenosine receptors widely expressed in the CNS, along with the receptor activation effect.

Purinergic Receptor	Localization in the CNS	Receptor Activation Effect	Reference
A_1_R	Presynaptic neurons	Inhibition of neurotransmitter release	[76]
	Postsynaptic neurons	Neuron depolarization	[76]
	Oligodendrocytes	Premature differentiation Promotion of myelination	[77,78]
	Astrocytes	Promotion of proliferation	[77]
A_2_R	Presynaptic neurons	Increase in neurotransmitter release	[76]
	Postsynaptic neurons	Increase in cellular excitability	[76]
	Astrocytes	Increase in glutamate release ^1^	[79,80]

^1^ Function reported by in vitro studies, previously attributed only to neurons.

## Data Availability

Reviewed data are contained within the article.
